# Early experience of Retrograde Intrarenal Surgery for Renal stone: A Descriptive Cross-sectional Study

**DOI:** 10.31729/jnma.4819

**Published:** 2020-07-31

**Authors:** Robin Joshi

**Affiliations:** 1 Department of Urology, Kathmandu Medical College and Teaching Hospital, Sinamangal, Kathmandu, Nepal

**Keywords:** *nephrolithotomy*, *percutaneous*, *nephrolithiasis*, *renal*, *retrograde*, *surgery*

## Abstract

**Introduction:**

Retrograde intrarenal surgery with improving skill and knowledge may be considered one of the first-line treatment options for removal of renal stones. The study is done to find the outcome of retrograde intrarenal surgery (RIRS) in patients with renal stone.

**Method:**

This descriptive cross sectional study was carried out on patients undergoing retrograde intrarenal surgery for renal stone at a tertiary care hospital December 2019 to March 2020. Ethical approval was taken from the institutional review committee (Ref. no.200120202). The Convenient sampling method was applied. Data was collected and analyzed in Statistical Package for the Social Sciences version 20.0. Point estimate at 95% confidence interval was calculated along with frequency and proportion for binary data.

**Results:**

Out of the 28 patients, the retrograde intrarenal surgery was successful in 27 (96.4%) cases. There were 16 (57.15%) females and 12 (42.86%) male patients with the mean age of 37.86±11.47 years. Most of the stones were in renal pelvis 18 (64.28%) followed by lower calyx 8(28.57%). The mean diameter of the stone was 11.47±3.33mm whereas most of the stones were on the right side 16 (57.15%). The mean hardness was 1155.21±265.34 Hounsfield units. Perioperative complications like failed access sheath placement in 2 (7.14%) cases, hematuria in 6 (21.43%) cases, fever in 6 (21.43%) cases, and septicemia in 4 (14.28%) cases.

**Conclusions:**

We found that the success rate of retrograde intrarenal surgery for the renal stone was acceptable and similar to other published studies . Retrograde intrarenal surgery is feasible for the treatment of kidney stones with acceptable complications and success rates.

## INTRODUCTION

The retrograde intrarenal surgery (RIRS) has become an effective tool in treating renal stones. Recent advances in technology like a thinner, better vision of the flexible scope, use of access sheath, stone basket and better learning curve of Urosurgeons in adapting to this new technique have led to better management of kidney stones.

Although, percutaneous nephrolithotomy (PCNL) is still the first-line treatment of renal stones of more than 2 cm with a high clearance rate but has increased morbidity and longer hospital stay as compared to RIRS.^1–4^ It has also been proposed to be a safe method in patients with co-morbid conditions like cardiopulmonary disease and advanced age. This procedure can also be effectively used in cases with abnormal anatomy.^5,6^

Therefore, this descriptive cross-sectional study was carried out to find out the success postoperative and outcomes of retrograde intrarenal surgery (RIRS) in patients with renal stones.

## METHODS

A descriptive cross sectional study was carried out in the Department of Surgery of the Kathmandu Medical College and Teaching Hospital (KMCTH) December 2019 to March 2020. Ethical approval was taken from the institutional review committee (IRC) with the reference number (200120202). Patients undergoing RIRS for renal stones were included in the study. Patients with severe co-morbid conditions and stone more than 20mm were excluded from the study. All patients were informed about the potential outcomes of RIRS procedure and signed informed consent was taken. The convenient sampling method was applied and the sample size was calculated as,

$$\begin{array}{ll} \text{n} & \text{= }{\text{Z}}^{\text{2}}\times \text{p}\times (1-\text{p)}/{\text{e}}^{\text{2}}\\ & \text{= }{\left(1.96\right)}^{\text{2}}\times 0.9\times (1-0.9)/{(\text{0.12)}}^{\text{2}}\\ & \text{= }24 \end{array}$$

Where,

n = required sample size
p = prevalence of success after RIRS surgery, 90%
e = margin of error, 12%
Z = Confidence interval at 95%

Taking a non-respondent rate of 10%, the sample size was calculated to be 26.4. However, 28 patients were taken in the study.

Most of the patients were performed under spinal anesthesia. Holmium laser was used as a source for stone fragmentation using flexible(Wolf, cobra double lumen) scope via ureteral access sheath. Demographic variables like age, gender, co-morbid conditions were recorded. Size,number,Hounsfield unit of the stone were taken into consideration. Peri-operative data included operative time,the energy of laser pulses, hospital stay, readmission and complications. All patients except one were presented and RIRS was performed after 2-3 weeks time. Hydrophilic safety guide wire 0.0035 straight tip and another Flexi tip guidewire were inserted via a double-lumen ureteric catheter after retrograde pyelogram(RPG). Semi rigid ureteroscopy was done to verify vesicoureteric junction (VUJ) and the rest of the Ureter before the procedure.

Access sheath (12-14 or11-13fr) was introduced under fluoroscopy guidance upto upper Ureter. Cobra Flexible URS double-lumen by Wolf was used. Lower calyceal stones were either relocated by using Dormia basket or lazed there only whenever possible. Holmium laser (35 watts, ZENA) was used at 10-15 wt with 8-10 joules of energy. Stone was fragmented or dusted as per requirement. The invisibility of stone or residual stone less the 4 mm on fluoroscopy is considered a successful stone clearance of stone. Lazing was stopped when stone fragments are not visible or not seen in fluoroscopy. During the procedure, irrigation is done by free flow aided by a pathfinder pump. Access sheath was removed along with scope taking good look and care of mucosa. Patients were re-stented for 3-4 weeks. Xray of the kidney, ureter, and bladder(KUB) and ultrasound were done to assess the presence of stone before removal of the stent.

Modified Clavien classification was used to grade the complication. All complications were recorded and treated accordingly. DJ stent was removed after 3-4 weeks after evaluating with XrayKUB and Ultrasound for remaining stones. Stones ≤ 4mm was considered stone free. Patients were kept on regular follow up as required. Readmission for fever, burning micturition, hematuria was treated with removal of DJ Stent and pharmacological treatment.

Data analysis was done in the Statistical Package for the Social Sciences( SPSS,version 20.0, SPSS Inc., Chicago,IL,USA). Point estimate at 95% confidence interval was calculated along with frequency and proportion for binary data.

## RESULTS

Out of the 28 patients, the RIRS surgery was successful in 27 (96.4%) cases. Six (21.43%) were readmitted with complications like fever and hematuria for which the double J (DJ) stent was removed. All of them improved with further conservative treatment. There were 26 (92.85%) prestented cases. There were perioperative complications like failed access sheath placement in 2 (7.14%) cases, hematuria in 6 (21.43%) cases, fever in 6 (21.43%) cases, and septicemia in 4 (14.28%) cases. Modified Clavien score was found to be in II and IIIa. The mean operative time was 124.8±24.17 minutes, with a mean hospital stay of 3.35±1.44.days (Table 1).

**Table 1:** Perioperative characteristics.

Variable	n (%)
**Pre-stented**	
Yes	26 (92.85)
No	2 (7.14)
Mean Operative time	124.8±24.17.
**Peri-operative complications**	
Failed access sheath placement	2 (7.14)
Hematuria	6 (21.43)
Fever	6 (21.43)
Septicemia	4 (14.28)
Sepsis	0
Steinstrasse	0
**Removal of DJ stent**	
During admission days	10 (35.71)
After Discharge (due to fever)	6 (21.43)
Mean hospital stay (1-6 days)	3.35±1.44.
**Post-operative outcomes**	
Readmission	6 (21.43)
Retreatment	1 (3.5)
Success rate	27 (96.4)

There were 16 (57.15%) females and 12 (42.86%) male patients with the mean age of 37.86±11.47 years. Three (10.71%) patients presented with a history of hypothyroidism and 1 (3.57%) with hypertension. Two (7.14%) presented with some form of an anomaly. There wasthe previous history of treatment like extracorporeal shock wave lithotripsy (ESWL) in 2 (7.14%) (Table 2).

**Table 2:** Socio-demographic characteristics.

Variables	n (%)
**Sex**	
Male	12 (42.86)
Female	16 (57.15)
Mean Age	37.86±11.47
**Co-morbidity**	
Diabetes	0
Hypothyroidism	3 (10.71)
Hypertension	1 (3.57)
**Anomaly**	
Malrotation	1 (3.57)
Double Ureter	1 (3.57)
**Previous treatment**	
ESWL	2 (7.14)

Most of the stones were in renal pelvis 18 (64.28%) followed by lower calyx 8 (28.57%). The mean diameter of the stone was 11.47±3.33mm. whereas most of the stones were on the right side 16 (57.14%). The mean hardness was 1155.21±265.34. Hounsfield units (HU) with a range of 572 to 1801 HU. Twenty-six (92.85%) cases presented with a single stone (Table 3).

**Table 3:** Stone characteristics.

Variables	n (%)
**Site**	
Pelvis	18 (64.28)
Upper calyx	1 (3.5)
Mid calyx	1 (3.5)
Lower calyx	8 (28.57)
**Number of stones**	
Single	26 (92.85)
Multiple	2 (7.14)
**Laterality**	
Right	16 (57.14)
Left	12 (42.86)
**Properties**	
Mean stone diameter(mm)	11.47±3.33
Mean Hardness	1208±S.D. HU

## DISCUSSION

This is an era of minimally invasive techniques for stone diseases. PCNL is a very popular technique of stone treatment worldwide but is not without morbidity. Complication during PCNL is reported by up to 20%.^7^ RIRS has evolved with better scope and technique during the past few years. It is proving to be an effective and better less invasive technique for kidney stones.^8,9^ Development of new generation flexible ureteroscope with increased deflection have decreased morbidity with acceptable success rate.^10,11^

The Stone free rate after RIRS has been reported above 90% for renal stone and 85% for lower calyceal stone. Further evaluation of stone length and variation in the anatomy of lower calyces will differ stone clearance rate.^12^ Hyams et al. further found that 7% of low calyceal stones were inaccessible.^13^ Most of the other published articles have focused on mean size, location,Hounsfield unit of stone, operative time, retreatment with readmission and complication during RIRS. Hyams et al. and Saddik et al. reported on RIRS fora stone size of 2-3 cm.^14,15^ In our cases the mean size of the stone was 11.47±3.33mm.

The use of access sheath has been another area of debate. Access sheath facilitates easy passage of scope, may reduce intrarenal pressure protecting Ureter as well as the scope.^15–18^ We have used access sheath in all presented cases (99%). Presenting facilitates easy passage of access sheath with few failures(7.14%). Schoenthater et al.and Palmero et al. reported sheath insertion failure in 3.77%.^19,20^ Palmero et al. further assessed the use of semi-rigid ureteroscope to evaluate the easy insertion of access sheath and use of it also helped in further dilatation of vesicoureteric junction the intramural part of Ureter.^20^

The success rate was 73.6% (for a single procedure) and 93.4% for retreatment reported by Palmero et al. which was similar to Alqahtani (96.7%) and Breda etal.^6,18,20,21^ Many studies considered a stone size of 20 mm and some less than 9mm.^18,21,22^ Retreatment has been reported upto 1.6-1.7mm.^6^ Modified Clavien classification system was used to grade the complication rate. Palmero et al. found a complication rate of 6.7% with an overall complication of 10%.^6,16^ Likewise, Shuba De et al. found the rate of fever to be between 2-28% and sepsis of 3-5 %.^23^ During our initial cases, there wasa spike ofhigh-grade fever in many cases postoperatively which was attributed to intrarenal pressure during the procedure. This was proved by the fact that fever was not seen in latter cases after we started to use pathfinder to reduce the sudden or persistent rise of intrarenal pressure.

Hospital stay is strongly correlated with Clavien score, complication rate, co-morbidities and co-morbidities being the strong predictor of complication.^24^

The result of our study supports RIRS as a safe procedure for the treatment of stones ≤20mm. Our study showed a 96.4% success rate for a mean stone size of 11.47±.3.33mm. Mean hospital stay was 3.35±1.44. days with readmission at 21.43% in our cases. One patient required retreatment in our cases.

This study has been calculated in a single center with a limited sample size. Convenient sampling has been done to include patients in the study. Therefore, the study findings may not be generalized in all settings. A multi-centered study with a larger sample employing a study design of higher evidence should be carried out.

## CONCLUSIONS

Our study has found the success rate of RIRS for renal stone is acceptable and similar to other published studies . Therefore, we consider RIRS is a safe and effective procedure to treat kidney stones. However, inclusion of larger number of cases and clinical trials must be carried out for better statistical value and support the evidence.

## Conflict of Interest

**None.**
